# The impact of mild induced hypothermia on the rate of transfusion and the mortality in severely injured patients: a retrospective multi-centre study

**DOI:** 10.1186/s40001-016-0233-x

**Published:** 2016-10-06

**Authors:** Kai Oliver Jensen, Leonhard Held, Andrea Kraus, Frank Hildebrand, Philipp Mommsen, Ladislav Mica, Guido A. Wanner, Peter Steiger, Rudolf M. Moos, Hans-Peter Simmen, Kai Sprengel

**Affiliations:** 1 Division of Trauma Surgery, University Hospital Zurich, University of Zurich, Raemistrasse 100, 8091 Zurich, Switzerland; 2 Department of Orthopedic Trauma, University of Aachen, Aachen, Germany; 3 Trauma Department, Hannover Medical School, Hannover, Germany; 4 Department of Biostatistics, Institute for Epidemiology, Biostatistics and Prevention, University of Zurich, Zurich, Switzerland; 5 Division of Surgical Intensive Care Medicine, University Hospital Zurich, University of Zurich, Zurich, Switzerland; 6 University Hospital Zurich, University of Zurich, Zurich, Switzerland

**Keywords:** Severely injured, Induced hypothermia, Transfusion rate, Mortality

## Abstract

**Background:**

Although under discussion, induced hypothermia (IH) is an established therapy for patients with cardiac arrest or traumatic brain injuries. The influences on coagulopathy and bleeding tendency in severely injured patients (SIP) with concomitant traumatic brain injury are most widely unclear. Therefore, the aim of this study was to quantify the effect of mild IH in SIP with concomitant severe traumatic brain injuries on transfusion rate and mortality.

**Methods:**

In this retrospective multi-centre study, SIP from three European level-1 trauma centres with an ISS ≥16 between 2009 and 2011 were included. At hospital A, patients qualified for IH with age ≤70 years and a severe head injury with an abbreviated injury scale (AIS_Head_) of ≥3. IH was defined as target core body temperature of 35 °C. Hypothermic patients were matched with two patients, one from hospital B and one from hospital C using age and AIS_Head_. The effect of IH on the transfusion rate, complications and mortality was quantified with 95 % confidence intervals (CI). Patients not treated with IH in hospital A and those from hospital B and C, who were not matched, were used to adjust the CI for the effect of inter-hospital therapy protocol differences.

**Results:**

Mean age of patients in the IH-group (*n* = 43) was 35.7 years, mean ISS 30 points and sex distribution showed 83.7 % male. Mean age of matched patients in the normotherm-group (*n* = 86) was 36.7 years, mean ISS 33 points and there were 75.6 % males. For the hypothermic patients, we pointed out an estimate of mean difference for the number of transfused units of packed red blood cells as well as for mortality which does not indicate a decrease in the benefit gained by hypothermia. It is suggested that hypothermic patients tend to a higher rate of lung failure and thromboembolisms.

**Conclusion:**

Though tending to an increased rate of complications, there is no evidence for a difference in both; rate of transfusion and mortality in SIP. Mild IH as an option for severe head injuries seems as well-being practicable in the presence of multiple severe injuries. Further, clinical studies regarding the side effects are necessary.

## Background

Hypothermia is classified by causation and extent. Causation of hypothermia can be endogen, controlled-induced or accidental [1–3]. The extent of hypothermia is mild (<35–34 °C), moderate (34–32 °C) or severe (<32 °C) [1]. Moderate controlled-induced hypothermia is an established therapy for patients with cardiac arrest in the post-resuscitation phase [4]. Guidelines suggest that a temperature of 32–34 °C should be applied in pre-hospital as well as in inner-hospital management of cardiac arrest, although Nielsen et al. found out that unconscious survivors after cardiac arrest do not benefit from a targeted temperature of 33 °C in contrast to 36 °C [4–6]. Hypothermia appears to have benefits on elevated intracranial pressure (ICP) in patients with traumatic brain injury within the first 48 h post-trauma if a temperature of 35 °C is applied with external surface cooling or with intra-vascular devices. Patients treated with hypothermia were less likely to die or to have an unfavourable outcome than those in the control group [7, 8]. On the other hand, it is common knowledge that accidentally occurring hypothermia seems to be correlated with a propensity for bleeding complications without being an independent predictor of mortality [9]. Accidental hypothermia, acidosis and coagulopathy represent the lethal triad in severely injured patients [10]. In the current literature, we found no studies in which permissive hypothermia or IH was investigated concerning severely injured patients with concomitant traumatic brain injury. Most studies deal with accidental hypothermia, IH in patients with cardiac arrest or animal trauma models treated with IH [1, 4, 9, 11, 12]. There is thus a lack of information about the effects of IH for severely, multiple injured patients with more than one injured body region. The aim of this study was to highlight the impact of mild IH of 35 °C in these severely injured patients with respect to the transfusion rate within the first 48 h and the in-hospital mortality.

## Patients and methods

### Study design and hypothesis

We conducted a retrospective multi-centre study to investigate the effect of mild IH in severely injured patients, with a target core temperature of 35 °C on the transfusion rate within the first 48 h post-trauma and on in-hospital mortality in comparison to matched normotherm patients.

### Patients

All patient data were prospectively collected for the internal trauma databases from three European University level-1 trauma centres, hereafter called hospital A, hospital B and hospital C. Inclusion criteria were severely injured patients, defined by an ISS ≥16 including severe head injury, defined by an AIS_Head_ ≥3, admission within the period from January 2009 to December 2011 and need for an initial intensive care treatment after damage control surgery [13]. Excluded were patients with restricted comfort therapy due to declared intention, age <18 years, an early relocation within the first 48 h and a late admission of ≥4 h counted from time of injury.

### Methods

#### Cooling

**Fig. 1:**
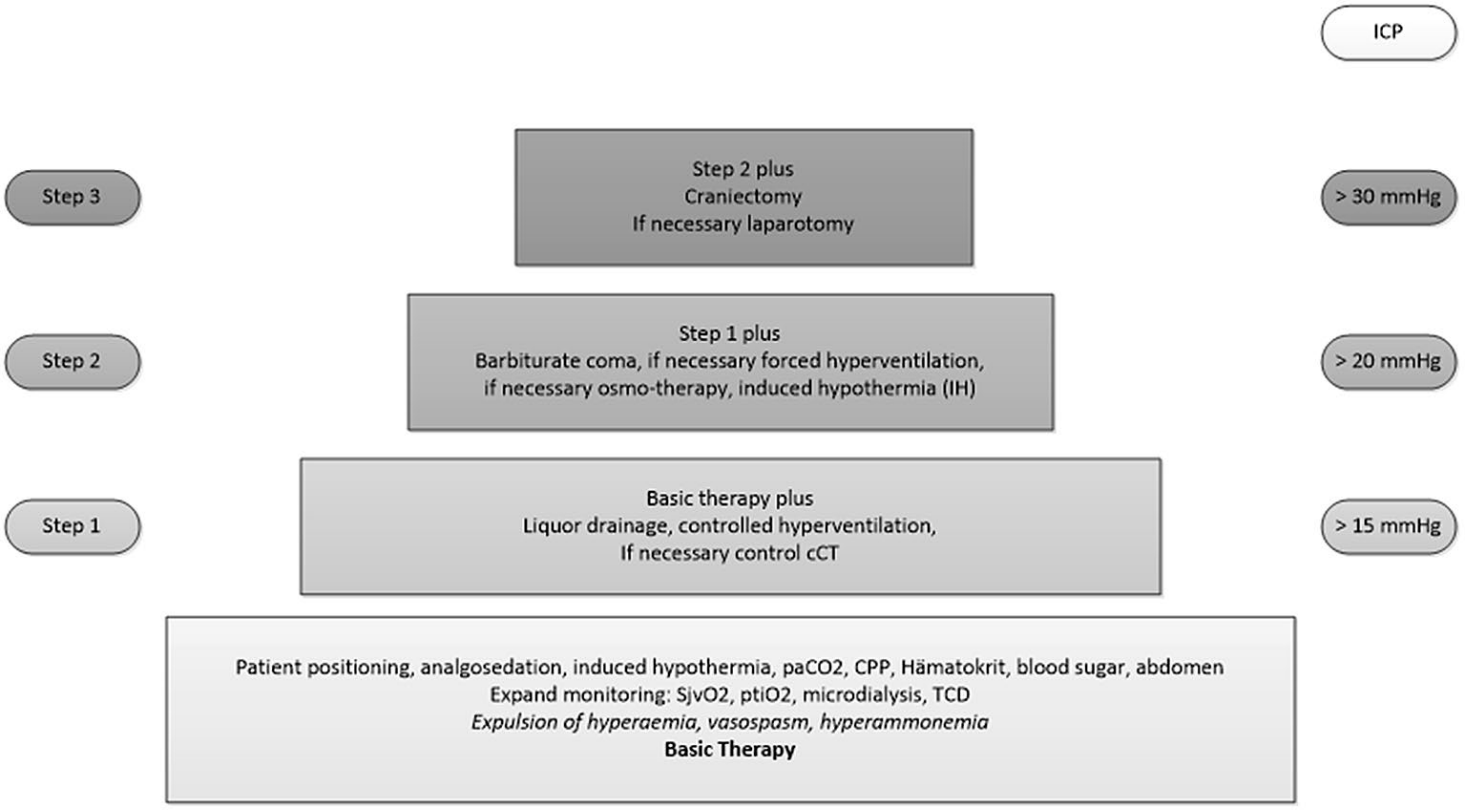
Step-based algorithm for treatment of traumatic brain injuries in hospital A according to the ICP

In hospital A, hypothermia is induced using a clinically approved algorithm for traumatic brain injuries, although other injuries may be present (Fig. 1). The intended temperature for hypothermia in hospital A was 35 °C. Down-regulation of the temperature was accomplished by a regime with different phases: (1) removal of blanket; (2) Coolmat (Blanketrol® II Hyper-Hypothermia System, Cincinnati Sub-Zero Products Inc., Cincinnati, USA); (3) Coolgard® (Femoral or subclavian vein) (Zoll CoolLine catheter, Zoll Circulation Inc., Sunnyvale, USA). The minimum duration of hypothermia was specified as 24 h. Within the protocol of hypothermia, deep analgosedation was performed. In hospitals B and hospital C, the aim of temperature management was normothermia. Within this study none of the patients had to be cooled via Coolgard®.

### Measurement of body temperature

The temperature was measured in a standardised manner depending on availability. If available a probe for continuous monitoring of brain tissue oxygen and brain temperature was installed (Neurovent-P-Temp, Raumedic AG, Helmbrechts, Germany). Following a descending order, the ocular surface temperature, measurements via pulse contour cardiac output (PiCCO) (Picco 2, Pulsion Medical Systems SE, Feldkirchen, Germany) or aural temperature probe were used.

### Definition of organic failure

Organic failure (OF) is defined as at least one organ scoring three or more points according to the Sequential Organ Failure Assessment (SOFA) score, matching the following criteria: *cardio circulatory system* dopamine greater than 5 µg/kg per minute or any adrenaline/noradrenaline treatment; *lung* Horowitz score (PaO2/FiO2) of less than 200 mmHg; *liver* serum bilirubin level of ≥6.0 mg/dL; *kidney* serum creatinine levels of 3.5 mg/dL or greater or urine output of less than 500 cc/day; *CNS* Glasgow Coma Scale (GCS) of 8 points or less [14]; multi-organ failure (MOF) is defined by the simultaneous failure of two or more organ systems. Sepsis is defined by the ACCP/SCCM Consensus Conference criteria [9, 15].

### Variables

Primary outcome variables were the number of transfused units of packed red blood cells (EC) and mortality. In addition, we recorded number of transfused units of fresh-frozen plasma (FFP) and thrombocyte units (TC) in cc. Secondary outcome variables such as organic failure (OF) distinguished by relevant organs, MOF, the occurrence of sepsis and thromboembolism (TE) were recorded as well. For the primary outcome variables, a length of stay (LOS) in hospital of 48 h or more was required, whilst for secondary outcome variables, an LOS in hospital of at least 7 days without relocation was necessary.

### Matching and statistical analysis

During the evaluation period, in hospital A hypothermia was induced as a standard of care in patients when aged ≤70 years and suffering from a severe head injury with an abbreviated injury scale (AIS_Head_) of ≥3. In consecutive absence of an inner-hospital control group, we matched patients treated with IH with one patient of hospital B and one patient from hospital C according to the characteristics, that determined the qualification for hypothermia in hospital A [16].

We estimated the effect of IH by means of a 95 % confidence interval (CI). For continuous outcomes (e.g. EC, FFP, TC), we computed non-parametric bootstrap CIs for the mean difference between the outcomes of the matched patients [17]. For binary outcomes (e.g. death), we constructed Wald CI for the log odds ratio, using conditional logistic regression. To explore the difference in outcome due to inter-hospital therapy protocol differences and to reduce the risk of a possible selection bias, we estimated the hospital effect with 95 % CI using the outcomes of patients who were not treated with IH in hospital A as well as for patients from hospitals B and hospital C, who were not matched to the patients treated with IH in hospital A. In this analysis, we adjusted for the age and ISS of the patient. Finally, the estimated hospital effect was subtracted from the estimated effect of hypothermia computed in the first step, and the two CIs were combined using the square-and-add approach, to obtain CI for the effect of IH [18]. Confidence intervals for log odds ratios were transformed to the risk difference scale assuming a baseline mortality equal to the average mortality of the patients from hospitals B and hospital C matched to the patients from hospital A. The analysis was performed separately for the two hospital pairs (A vs. B and A vs. C). Statistical analysis has been performed in the R programming language [19].

## Results

**Fig. 2:**
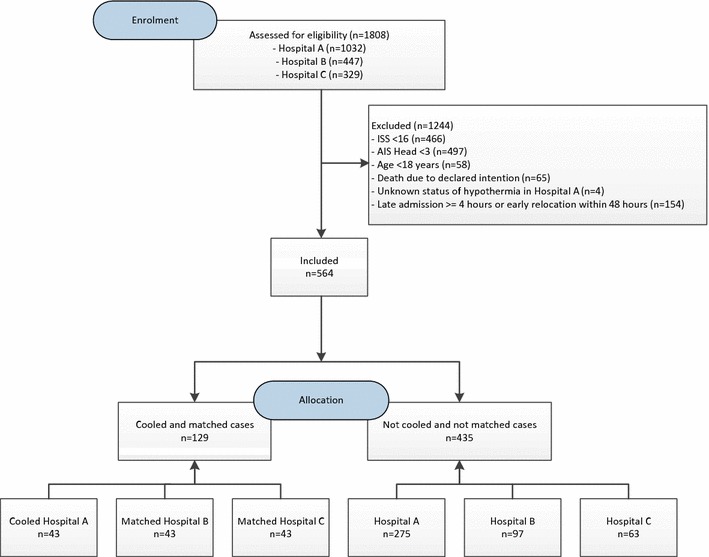
Flow diagram of enrolment and allocation

A total of 1808 patients were evaluated in this study. Regarding the underlying inclusion and exclusion criteria 564 patients were included, of whom 43 patients were treated with hypothermia in hospital A. A data set was created with 129 patients including the 43 hypothermic patients in hospital A as well as the matched normotherm patients from hospital B and hospital C (*n* = 86) (Fig. 2).

The mean ISS of the hypothermic patients was 30 points (SD 9.6 points), mean age was 35.7 years (SD 13.2 years) with 83.7 % males. The mean ISS of the matched normothermic patients was 33 points (SD 12.4 points), mean age was 36.7 years (SD 13.3 years) and 75.6 % patients were males. Base data for each hospital are shown in Table 1 separately. AIS distribution regarding the main six regions of interest (head; face; thorax; abdomen; extremities; external) is demonstrated in Table 2. In mean, 60.5 % of the hypothermic and matched patients suffered from a relevant injury (AIS ≥ 3) of other regions than AIS_Head_.

**Table 1:** Base data separately for each hospital divided into hypotherm, matched normotherm and remaining cases of normotherm population

	Hospital					
	A	B	C	A	B	C
	Hypotherm	Matched	Matched	Normotherm		
Variable	*n* = 43	*n* = 43	*n* = 43	*n* = 275	*n* = 97	*n* = 63
Age (years)	36 ± 13	38 ± 13	36 ± 14	55 ± 22	50 ± 23	61 ± 18
ISS	30 ± 10	33 ± 10	33 ± 14	28 ± 10	27 ± 8	27 ± 10
NISS	44 ± 14	43 ± 13	43 ± 18	37 ± 13	32 ± 12	36 ± 14
TRISS	0.68 ± 0.28	0.73 ± 0.26	0.64 ± 0.36	0.71 ± 0.31	0.69 ± 0.31	0.61 ± 0.33
Blunt trauma mechanism (%)	97.6	95.1	100	95.6	97.9	98.4
Sex female (%)	16.3	27.9	18.6	33.8	34.0	25.4
At arrival in resuscitation room						
SAP (mmHg)	130 ± 26	115 ± 32	116 ± 40	135 ± 32	124 ± 31	127 ± 42
Heart rate (beats/min)	87 ± 22	94 ± 25	82 ± 31	87 ± 22	86 ± 23	90 ± 25
Temperature (°C)	35.3 ± 1.2	35.1 ± .13	34.7 ± 2.4	35.2 ± 1.4	35.4 ± 1.2	35.6 ± 2.1
Haemoglobin (g/L)	12.5 ± 1.8	11.6 ± 2.7	13.1 ± 2.2	12.1 ± 2.5	11.5 ± 2.6	12.0 ± 2.9
Thrombocytes (10^3^/µL)	218 ± 57	194 ± 60	216 ± 72	224 ± 80	198 ± 62	214 ± 73
Prothrombin time (%)	80 ± 18	71 ± 25	71 ± 25	74 ± 25	72 ± 21	78 ± 20
Base excess (mmol/L)	−3.3 ± 2.8	−3.4 ± 5.2	−2.6 ± 4.2	−2.4 ± 4.7	−2.6 ± 3.7	−3.3 ± 4.3
Lactate (mmol/L)	2.3 ± 1.7	2.8 ± 2.0	3.6 ± 3.7	2.2 ± 1.8	2.6 ± 1.8	2.9 ± 2.6

Data given as mean and standard deviation, *ISS* injury severity score, *NISS* new ISS, *TRISS* trauma ISS, *SAP* systolic arterial pressure

**Table 2:** AIS distribution by hospital for hypothermic and normothermic matched patients

AIS region	Value	Hospital		
		A (*n*)	B (*n*)	C (*n*)
Head	3	3	3	3
	4	20	19	14
	5	20	21	26
Face	0	22	28	23
	1	4	6	5
	2	9	6	8
	3	8	3	6
	4	0	0	1
	5	0	0	0
	6	0	0	0
Thorax	0	21	9	25
	1	0	2	1
	2	4	10	6
	3	14	17	5
	4	4	3	4
	5	0	2	1
	6	0	0	1
Abdomen	0	31	29	33
	1	0	0	0
	2	11	12	1
	3	1	1	5
	4	0	1	4
	5	0	0	0
	6	0	0	0
Extremities	0	25	18	27
	1	0	1	1
	2	11	11	8
	3	6	9	5
	4	1	4	1
	5	0	0	1
	6	0	0	0
External	0	22	35	33
	1	14	6	4
	2	6	2	5
	3	1	0	0
	4	0	0	1
	5	0	0	0
	6	0	0	0

### Primary outcomes

**Fig. 3:**
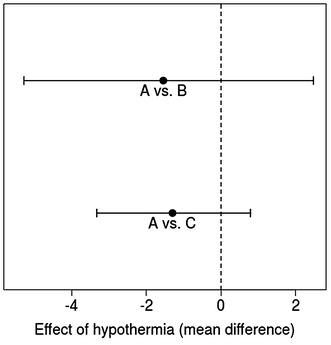
Mean difference and 95 % CI for EC as effect of hypothermia for hospitals *A vs. B* and *A vs. C*

**Fig. 4:**
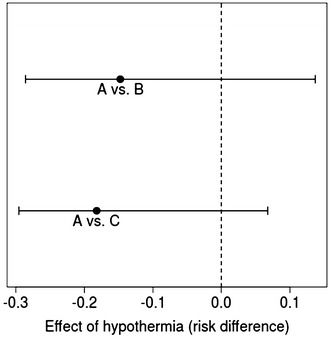
Risk difference and 95 % CI for in-hospital mortality as effect of hypothermia for hospitals *A vs. B* and *A vs. C*

Considering the rate of EC transfused within the first 48 h post-trauma, an estimated mean of −1.5 units [95 % CI −5.3 to 2.5 for hospital A in comparison to hospital B (AB)] and −1.3 units (95 % CI −3.3 to 0.8 AC) was calculated. We found an RD for mortality of −0.14 (95 % CI −0.29 to 0.14 AB) and −0.18 (95 % CI −0.30 to 0.07 for AC) (Figs. 3 and 4).

### Secondary outcomes

The existence of IH seems to have a negative side effect on the prevalence of organic failure of the lungs with OR of 4.7 (95 % CI 1.0–22.6 AB) and 4.7 (95 % CI 0.9–24.4 AC) and the occurrence of thromboembolism with OR of 12.9 (95 % CI 1.2–133.9 AB) and of 21.0 (95 % CI 2.0–217.4 AC) (Table 3).

**Table 3:** 95 % confidence intervals for all outcomes

Outcome parameter	Comparison	Estimate	95 % CI from	To
EC difference	AB	−1.5	−5.3	2.5
	AC	−1.3	−3.3	0.8
FFP difference	AB	1.2	−2.0	4.0
	AC	0.7	−1.7	2.8
TC difference	AB	−0.5	−0.9	0.3
	AC	−0.1	−0.3	0.2
Death (OR)	AB	0.5	0.1	1.8
	AC	0.4	0.1	1.3
Of lung (OR)	AB	4.7	1.0	22.6
	AC	4.7	0.9	24.4
TE (OR)	AB	12.9	1.2	133.9
	AC	21.0	2.0	217.4

*EC* number of transfused units of packed red blood cells, *FFP* number of transfused units of fresh-frozen plasma, *TC* number of transfused units of thrombocyte units, *OF* organic failure, *TE* thromboembolism, *CI* confidence interval, *AB* hospital A in comparison to hospital B, *AC* hospital A in comparison to hospital C, *OR* odds ratio

Due to the small number of specific complications, the OR for sepsis as well as for organic failure of liver and kidneys was not further taken into account. Organic failure of the heart and the central nervous system are secondary outcomes, which cannot be used for further comparison due to the hypothermia protocol used in hospital A, including the proposed deep analgosedation with a consecutive Glasgow Coma Scale of 3 and the common application of noradrenaline leading to an organic failure classification (central nervous system, heart) following the SOFA score [14]. The variable multi-organ failure defined as a combination of two or more organic failures is falsified as a sequella.

## Discussion

The present study indicates that mild IH of 35 °C neither has a negative effect on the coagulation system of severely injured patients measured by transfusion rate nor on the mortality. Actual literature suggests a benefit of 15 % for induced hypothermia [20]. Our data showed an upper margin of the 95 % CI within this range for mortality and the comparison between both hospital pairs. The same evidence was given for the amount of EC transfused. We could not prove an increase in bleeding complications or in-hospital mortality due to hypothermia.

Underlining our conclusions, an improvement of survival was measured in an uncontrolled haemorrhagic shock model comparing moderate hypothermia versus normothermia [21, 22]. Whilst another study ascribes significant coagulopathy in severely injured patients to hypothermia, it has only been shown for hypothermia below 34 °C. The authors presume hypercoagulability to partially compensate the restrictions of coagulation due to hypothermia [23]. These results do conform to the findings in the present study with an aim of 35 °C. In the same way, the mortality due to accidental hypothermia varies from 30 % up to 100 % depending on the depth of accidental hypothermia [2, 24, 25]. Accidental hypothermia seems to trigger the vicious circle of the lethal triad of hypothermia, acidosis and coagulopathy [10].

Based on our results, we agree with Mohr et al. who reported of an IH animal trauma model without an increase of coagulopathy. Within that study, there were no findings showing negative impact of hypothermia on inflammatory complications and mortality [12]. Other authors mention the negative effects of hypothermia without further exploring them [26].

Although our investigations suggested lung failure and TE to be bigger problems for patients treated with hypothermia, it is not associated with a higher mortality but only with morbidity. Related to these findings, Alderson et al. showed that hypothermia resulted in a statistically significant increase in the OR for incidence of pneumonia and a decrease in the OR for death in patients with head injuries, whereas Geurts et al. observe in a meta-analysis no increase in the overall risk of pneumonia or sepsis but strongly suggest an association between them [27, 28]. Descending into the matter of blood levels of TNF-alpha and IL-6 in hypothermic animals as inflammatory cytokine mediators, Gröger et al. found almost similar values at 35 °C and with normothermia in contrast to hypothermia of 32 °C [29]. In summary, the temperature seems to be mainly affecting the morbidity of lung failure and TE. Even though we expected an association between IH and morbidity, we could not show an increase of in-hospital mortality.

We are aware of some weaknesses in this study. The main handicap is the retrospective nature of the study, although all data were recorded prospectively. Additionally, a larger number of matched cases would provide greater confidence in the significance of the results. Furthermore, the absence of an inner-hospital control group due to the cooling-protocol in hospital A lead to this elaborate statistical design.

We conclude that severely injured patients did not suffer from relevant bleeding complications due to IH because of a similar transfusion rate.

## Conclusion

The combination of the potential positive effects of hypothermia for traumatic brain injuries and of not increasing the usage of blood products or the mortality makes mild controlled-induced hypothermia a feasible procedure for severely, multiple injured patients with concomitant, severe traumatic brain injury. We look forward to upcoming prospectively randomised controlled studies on this topic with great pleasure and interest.

## Abbreviations

IH: induced hypothermia
SIP: severely injured patients
ISS: injury severity score
AIS: abbreviated injury scale
CI: confidence interval
PiCCO: pulse contour cardiac output
SOFA: Sequential Organ Failure Assessment
GCS: Glasgow Coma Scale
MOF: multi-organ failure
EC: units of packed red blood cells
FFP: units of fresh-frozen plasma
OF: organ failure
TE: thromboembolism
LOS: length of stay
TC: thrombocyte units
SD: standard deviation
RD: risk difference or RISC difference
OR: odds ratio
cCT: cranial computed tomography
paCO2: arterial carbon dioxide pressure
CPP: cerebral perfusion pressure
SjvO2: jugular venous oxygen saturation
ptiO2: brain tissue oxygen
TCD: transcranial dopller
